# Associations between subjective overactive bladder symptoms and objective parameters on bladder diary and filling cystometry

**DOI:** 10.1007/s00192-012-1774-3

**Published:** 2012-04-28

**Authors:** N. M. P. Daan, K. J. Schweitzer, C. H. van der Vaart

**Affiliations:** Division of Woman & Baby, Department of Urogynecology, University Medical Center Utrecht, Heidelberglaan 100, 3584 CX Utrecht, The Netherlands

**Keywords:** Bladder diary, Cystometry, Overactive bladder

## Abstract

**Introduction and hypothesis:**

A study was conducted to assess associations between different overactive bladder (OAB) symptoms and their outcomes on bladder diary and filling cystometry parameters.

**Methods:**

We performed a retrospective cohort study in database of 6,876 Urinary Distress Inventories, 3,185 bladder diaries and 2,153 filling cystometries from women referred to our urogynecological center between 2003 and 2009. Women were dichotomized into two groups. Group I: those women without symptoms, and those with symptoms that were not bothersome. Group II: women with bothersome symptoms. Data obtained from bladder diaries were: daytime urinary frequency, nocturnal frequency, minimum voided volume, maximum voided volume, average voided volume, and incontinence episodes. From filling cystometries, volumes at first desire to void, normal desire to void, strong desire to void and maximum cystometric capacity, were extracted. Univariate and multiple linear regression analysis were performed to determine associations between OAB symptoms and bladder diary and filling cystometry measurements.

**Results:**

After multivariate analysis the objective daytime frequency was most strongly associated with the frequency symptom (β 0.27,* p* < 0.05), night time frequency with the nocturia symptom (β 0.40,* p* < 0.05) and the number of incontinence episodes with the urge incontinence symptom (β 0.37,* p* < 0.05). Both frequency and nocturia symptoms were significantly associated with bladder diary and cystometry filling volumes, and their effect size was the same. The urgency symptom proved to be poorly associated with objective parameters.

**Conclusions:**

In contrast to the frequency and nocturia symptom, the urgency symptom is poorly associated with objective parameters on bladder diary and filling cystometry. Therefore, the current practice of using frequency and incontinence episodes in outcome research of OAB trials is justified.

## Introduction

The overactive bladder syndrome (OAB) represents a group of prevalent micturition symptoms that are known to have a negative impact on quality of life [1–3]. Population-based studies on women report a prevalence of OAB that varies between 9.7% and 35.7%, with a substantial rise in prevalence with increasing age [4, 5]. Approximately 50% of women with OAB reported experiencing symptoms that were bothersome in their daily life. The OAB syndrome is internationally defined as: urinary urgency, usually accompanied by frequency and nocturia with or without urgency urinary incontinence, in the absence of urinary tract infection or other obvious pathology [6]. This definition indicates that the presence of urgency is mandatory in diagnosing OAB. However, in research on the outcome of treatment modalities for OAB, the primary outcome in the majority of studies is the reduction in daily micturition and/or urinary incontinence episodes. [7–10]. Apparently there is a discrepancy between the importance of the urgency symptom in making a diagnosis of OAB, and in the evaluation of OAB treatment. This raises the question whether the severity of the urgency symptom is quantitatively associated with objective parameters as measured with a bladder diary and filling cystometry.

The aim of our study is to assess the association between bothersome OAB symptoms and objective bladder diary and filling cystometry measures.

## Materials and methods

### Study population

A retrospective cohort study was performed with a database containing 6,876 questionnaires that were completed by women who were referred to our urogynecological center between 2003 and 2009. From this database our final study population comprised 3,280 women with 48-h bladder diaries and 2,153 women with available information on filling cystometries. This study was reviewed by our Medical Research Ethics Committee (MREC), which concluded that no Medical Research Involving Human Subjects Act (WMO) approval was needed, since the study neither implied that the women would receive a particular treatment, nor impacted on the behavior of the women.

### Measurements

As part of the routine work-up, all women filled out the validated Dutch version of the Urinary Distress Inventory (UDI) [11]. The original UDI consists of 19 items. Each item measures if a micturition symptom is present and to what extent the patient is bothered by this symptom. Bother is measured on a four-point Likert scale ranging from not at all to greatly bothered. For the purpose of this study we selected four lower urinary tract symptoms (LUTS) from the UDI that are in concordance with ICS definitions. LUTS were assessed with the following questions: on frequency *“Do you experience frequent urination?”*, on urgency *“Do you experience a strong feeling of urgency to empty your bladder?”*, on nocturia *“Do you experience frequent urination at night?”*, on urgency incontinence *“Do you experience urine leakage related to a feeling of urgency?”* [12].

We dichotomized the study population for every OAB symptom into two groups. Group 1 consisted of women without the symptom, or those with the symptom but without any bother. Group 2 consisted of women who were slightly, moderately or greatly bothered by one of the symptoms.

Women were asked to complete a 24-h bladder diary during a minimum of 2 days, prior to their consultation at our center. From these dairies, time and volume (measured with a measuring cup) of voiding episodes, as well as time and amount of fluid intake were recorded. The frequency and amount of incontinence were noted along with complaints of pain, urgency, straining to void, and bandage changes. From the bladder diaries we extracted the following information: daytime urinary frequency, nocturnal frequency, minimum voided volume, maximum voided volume, mean voided volume, and incontinence episodes.

Filling cystometry (Medtronic Duet®; Medtronic, Minneapolis, MN, USA) was performed at an infusion rate of 50 mL/min. Bladder sensations during filling cystometry were recorded, conforming to official ICS guidelines [6]. We did not collect all information about drug use, which might influence bladder function. In case of performing cystometry, all women stopped using anticholinergic drugs for at least one week prior to the investigation. Finally, we collected data on age and parity.

### Statistical analysis

The frequencies of reported OAB symptoms, mean age, and parity of women were assessed. A Student’s* t* test was used to compare mean bladder diary and filling cystometry parameters between groups. The chance of finding statistically, but not clinically significant differences increases with a large study sample. In order to assess the strength of the relationship, effect sizes (Cohen’s d) were calculated for all statistically significant differences (*p* < 0.05). An effect size between 0.2 and 0.4 was considered small, between 0.5 and 0.7 medium, and ≥0.8 as large [13]. Those OAB symptoms that proved to be significantly associated with bladder diary and filling cystometry parameters at a* p* value <0.10, were entered into a linear multiple regression analysis, with the bladder diary and cystometry items as dependent variables. The factor age was also included in multiple regression analysis. A subanalysis was performed on the data of women who also underwent a cystometry. As stated, none of these women used anticholinergic drugs. All statistics were performed using SPSS version 15.0.

## Results

There were 3,280 bladder diaries and 2,153 filling cystometries available for analysis. Ninety-five bladder diaries were excluded, because the women did not report any LUTS. The mean age of the studied population was 54.8 years (±14.7 years) and 85.2% were parous women. The prevalence of reported OAB symptoms in our study population is shown in Table 1. The vast majority of women (92%) experienced more than one bothersome lower urinary tract symptom. The combination of bothersome urgency, frequency, and nocturia was reported by 34.4% of women, and 26.2% of women additionally experienced urgency incontinence.

**Table 1:** Prevalence of bothersome overactive bladder (OAB) symptoms

OAB symptom	Women,* n* (%)
Frequency	2,349 (73.8)
Urgency	2,153 (67.6)
Nocturia	1,536 (48.2)
Urge incontinence	1,929 (60.6)

The data shown were calculated using the UDI questionnaires

The results of the univariate analysis (unpaired samples* t* test) are shown in Tables 2 and 3. The presence of bothersome OAB symptoms showed a statistically significant (*p* < 0.05) association with almost all bladder diary and filling cystometry parameters. If we consider an effect size ≥0.8 to be clinically relevant, the frequency symptom is best associated with daytime frequency, the nocturia symptom with nocturnal frequency, and the urgency incontinence symptom with incontinence episodes. Bladder diary and filling cystometry recorded filling volumes were significantly smaller in women with bothersome OAB symptoms than in women without bothersome OAB symptoms. The strongest association regarding volume measurements and OAB symptoms was found between frequency of voiding and the mean voided volume. Although statistically significant associations were found for the urgency symptom, the effect sizes were small, which may indicate little clinical relevance. Subanalysis of bladder diaries that were completed by women without anticholinergic therapy did not alter any of the results (data available on request).

**Table 2:** Mean outcomes with regard to bladder diary parameters for each OAB symptom

OAB symptom	Bladder diary measures	Group 1, mean (standard deviation)	Group 2, mean (standard deviation)	Cohen’s *d*
Frequency	Daytime frequency episodes	7.2 (2.2)	9.4 (3.1)	−0.82*
	Nocturnal frequency episodes	0.8 (0.9)	1.4 (1.2)	−0.59*
	Incontinence episodes	2.3 (3.0)	3.6 (4.0)	−0.37*
	VOL minimum	114 (78)	85 (58)	0.42*
	VOL mean	275 (108)	215 (88)	0.61*
	VOL maximum	507 (205)	432 (187)	0.39*
Urgency	Daytime frequency episodes	7.9 (2.5)	9.2 (3.2)	−0.45*
	Nocturnal frequency episodes	1 (1.0)	1.4 (1.2)	−0.32*
	Incontinence episodes	2.8 (3.3)	3.5 (4.0)	−0.22*
	VOL minimum	101 (70)	90 (63)	0.17*
	VOL mean	253 (105)	222 (92)	0.32*
	VOL maximum	486 (205)	436 (188)	0.26*
Nocturia	Daytime frequency episodes	8.1 (2.7)	9.5 (3.2)	−0.45*
	Nocturnal frequency episodes	0.7 (0.8)	1.9 (1.3)	−1.05*
	Incontinence episodes	2.8 (3.2)	3.8 (4.3)	−0.28*
	VOL minimum	103 (72)	83 (57)	0.43*
	VOL mean	256 (103)	205 (84)	0.55*
	VOL maximum	492 (200)	409 (180)	0.44*
Urge incontinence	Daytime frequency episodes	8.6 (3.1)	8.8 (3.0)	−0.07**
	Nocturnal frequency episodes	1.2 (1.2)	1.3 (1.2)	−0.08*
	Incontinence episodes	1.4 (2.6)	4.5 (3.9)	−0.92*
	VOL minimum	96 (71)	92 (62)	0.06**
	VOL mean	241 (103)	227 (94)	0.14*
	VOL maximum	462 (199)	447 (192)	0.07*

Group 1: women without symptoms, or symptoms that were not at all bothersome

Group 2: women with bothersome symptoms

VOL: volume in milliliters

*p < 0.05

**p ≥ 0.05 and <0.10

**Table 3:** Mean outcomes on filling cystometry parameters for each OAB symptom

OAB symptom	Filling cystometry measures	Group 1, mean (standard deviation)	Group 2, mean (standard deviation)	Cohen’s d*
Frequency	VOL first desire to void	235 (133)	196 (120)	0.31
	VOL normal desire to void	332 (144)	275 (141)	0.40
	VOL strong desire to void	495 (171)	412 (167)	0.49
	VOL maximum cystometric capacity	555 (171)	473 (168)	0.48
Urgency	VOL first desire to void	229 (135)	195 (118)	0.27
	VOL normal desire to void	320 (148)	275 (140)	0.31
	VOL strong desire to void	467 (176)	417 (168)	0.29
	VOL maximum cystometric capacity	527 (176)	479 (168)	0.28
Nocturia	VOL first desire to void	221 (127)	191 (120)	0.24
	VOL normal desire to void	309 (148)	271 (137)	0.24
	VOL strong desire to void	469 (173)	398 (163)	0.42
	VOL maximum cystometric capacity	534 (172)	455 (164)	0.47
Urge incontinence	VOL first desire to void	213 (127)	202 (124)	0.09
	VOL normal desire to void	306 (144)	281 (143)	0.17
	VOL strong desire to void	457 (172)	421 (170)	0.21
	VOL maximum cystometric capacity	520 (171)	480 (171)	0.23

Group 1: women without symptom, or with symptoms that were not at all bothersome

Group 2: women with bothersome symptoms

VOL: volume in milliliters

*p < 0.05

The results of the multiple regression analysis are listed in Table 4. After multivariate analysis for other OAB symptoms, the factor that was most strongly associated with bladder diary daytime frequency was the frequency symptom. The standardized β coefficients indicate that this effect was two to five times higher than the effect of other OAB symptoms. Frequency and nocturia are most strongly associated with voided volumes recorded using the bladder diary and filling cystometry. After multivariate analysis, urgency and urgency incontinence symptoms were no longer significantly associated with most bladder diary and filling cystometry parameters. Female age was evidently associated with nocturnal frequency episodes and volumes at first and normal desire to void.

**Table 4:** Results of multiple regression analysis from bladder diary and filling cystometry parameters

	Standardized β coefficients									
	Bladder diary measures						Filling cystometry measures			
	DF	NF	VOL min	VOL max	VOL mean	IE	FDV	NDV	SDV	MCC
Frequency	0.27*	0.10*	−0.17*	−0.11*	−0.20*	0.06*	−0.09*	−0.11*	−0.14*	−0.13*
Urgency	0.08*	0.02	0.01	−0.05*	−0.03	−0.01	−0.08*	−0.08*	−0.05	−0.04
Nocturia	0.13*	0.40*	−0.11*	−0.17*	−0.19	0.07	−0.11*	−0.11*	−0.16*	−0.18*
Urge incontinence	−0.05*	−0.04	0.01	0.01	−0.01	0.37*	−0.02	−0.05*	−0.05*	−0.06*
Age	−0.05*	0.17*	0.05*	0.01	0.01	0.05*	0.18*	0.17*	0.07*	0.04
Correlation coefficient (Adjusted R^2^)	0.13 *	0.26*	0.05*	0.06*	0.11*	0.17*	0.06*	0.07*	0.07*	0.08*

DF: daytime frequency, NF: nocturnal frequency, VOLmin: minimum voided volume, VOLmax: maximum voided volume, VOL mean: mean voided volume, IE: incontinence episodes, FDV: volume at first desire to void, NDV: volume at normal desire to void, SDV: volume at strong desire to void, MCC: volume at maximum cystometric capacity

*p < 0.05

## Discussion

The aim of the current study was to assess the association between the OAB symptoms and objective parameters from a bladder diary and filling cystometry. Of all four OAB symptoms the frequency symptom showed the strongest association with daytime urinary frequency, nocturia with nocturnal frequency, and urgency incontinence with incontinence episodes as measured in the bladder diary. The frequency and nocturia symptoms showed almost equal and strong associations with bladder volumes as measured in the bladder diary and by filling cystometry. The key symptom of the OAB syndrome, urgency, was either not at all or only poorly associated with objective parameters from the bladder diary and filling cystometry.

The urgency symptom has been adopted as the key symptom of the OAB syndrome. However, frequent day-time or night-time micturition may be perceived by women as bothersome, even in the absence of urgency [14, 15]. Furthermore, studies about health-related quality of life in women with OAB report that the presence of urgency alone is only associated with a moderate degree of bother [16]. Ever since the introduction of the OAB syndrome into official standardized terminology, there has been an ongoing debate about the accuracy and content of the definition of OAB. The emphasis on urgency as the key symptom is not based on a scientific –factor and may have originated from the introduction of the OAB syndrome by the pharmaceutical industry [17, 18]. The urgency symptom is notoriously difficult to define for both patient and expert. Although the definition appears quite simple, it is suggested that urgency might not be an “all or nothing” sensation. It is plausible that there are different types of urgency that can be experienced and modulated in various ways [19]. The individual perception of urgency makes it almost impossible to create a uniform definition for the heterogeneous patient population with OAB symptoms. Because of the subjective nature of the urgency symptom it is to be expected that associations with objective parameters are limited. Our results support this view. In many trials that evaluate the treatment of OAB, frequency and incontinence episodes, and not urgency, are used as primary outcome measures [7–10]. Our results support the use of frequency and incontinence episodes as outcome parameters in OAB studies, since these symptoms show the best associations between subjective and objective parameters.

It has also been previously demonstrated that the occurrence of the OAB syndrome is associated with objective parameters from the bladder diary and filling cystometry [20, 21]. However, these studies have not assessed which of the symptoms contained in the official definition of the OAB syndrome are responsible for this effect. Our results indicate that, after correction for the presence of other symptoms, the association between OAB and objective parameters is mostly attributable to the voiding frequency and nocturia. As stated, this may reflect the difficulty in defining and quantifying urgency.

One limitation of the current study may be due to the differences in the definition of urgency. The UDI definition of urgency that we used is: *“Do you experience a strong feeling of urgency to empty your bladder?”*. The current ICS definition of urgency is: *“Complaint of a sudden, compelling desire to pass urine which is difficult to defer”* [6]. These two definitions are not identical and therefore may not be interchangeable. With respect to urgency incontinence our definition is symptom-based. Therefore, we cannot be sure that women with stress incontinence at maximum cystometric capacity responded positively to the urgency incontinence question.

Another possible limitation of our study is the fact that the study population only consisted of women with urogenital dysfunction. Consequently, the women without a particularly bothersome OAB symptom that were entered into the comparison group (Group 1) were not entirely without symptoms, but did experience other symptoms of urogenital dysfunction. This may have limited the differences we observed between groups. However, despite this, the observed differences were not only statistically significant, but also clinically relevant as shown by our effect size statistics. If we had used a completely healthy control group, the differences were likely to have been even more pronounced.

Finally, we used a 48-h bladder diary, which is known to be less accurate than a bladder diary that covers 3–7 days [22]. However, it has been demonstrated that a prolonged duration of bladder diary recording is associated with lower compliance and higher burden for the patient [23, 24].

## Conclusion

Daytime and nocturnal frequency symptoms are best associated with objective frequency and volume parameters from a bladder diary and filling cystometry. In contrast, the urgency symptom, which is essential in the current definition of OAB, is poorly associated with these objective parameters. Therefore, the current practice of using frequency and incontinence episodes in the outcome research of OAB trials is justified.
